# Low genetic diversity but strong population structure reflects multiple introductions of western flower thrips (Thysanoptera: Thripidae) into China followed by human‐mediated spread

**DOI:** 10.1111/eva.12461

**Published:** 2017-02-23

**Authors:** Li‐Jun Cao, Ze‐Hua Wang, Ya‐Jun Gong, Liang Zhu, Ary Anthony Hoffmann, Shu‐Jun Wei

**Affiliations:** ^1^ Institute of Plant and Environmental ProtectionBeijing Academy of Agriculture and Forestry SciencesBeijingChina; ^2^ School of BioSciencesBio21 InstituteThe University of MelbourneMelbourneVic.Australia

**Keywords:** *Frankliniella occidentalis*, invasive species, microsatellite, mitochondrial gene, multiple introductions, population genetics

## Abstract

Historical invasion scenarios based on observational records are usually incomplete and biased, but these can be supplemented by population genetic data. The western flower thrips (WFT), *Frankliniella occidentalis*, invaded China in the last 13 years and has rapidly become one of the most serious pests in the country. To assess whether this invasion involved a single event or multiple events, we examined patterns of genetic diversity and population structure of WFT across 12 Chinese populations and a native US population based on mitochondrial DNA and/or 18 microsatellite loci. The average allelic richness and haplotype diversity in Chinese populations were significantly lower than in a population from its native range. The distribution of mitochondrial haplotypes suggested multiple independent invasions of WFT into China, including two invasions into the Beijing region. Based on microsatellite data, two distinct clusters were identified, with both of them splitting further into two clusters; in the Beijing region, the microsatellite data also provided evidence for two introductions. Both the absence of isolation by distance and the fact that distant populations were similar genetically suggest patterns of WFT movement linked to human activities. Our study therefore suggests multiple introductions of WFT into China and human‐assisted spread.

## 1 Introduction

Biological invasions, as by‐products of travel and trade, are becoming a worldwide environmental, health and agricultural issue (Pimentel et al., 2001; Ricciardi, 2007). Although many invasion events recorded in the literature are based on sample collections, introductions and dispersal routes are often undetectable by direct observations, particularly when introductions have taken place following failure in quarantine (Estoup & Guillemaud, 2010). Studies on the genetic diversity and population structure of an invasive species in both its introduced and native area can help to elucidate the source, path and times of introduction (Boissin et al., 2012; Lombaert et al., 2014; Rollins, Woolnough, Wilton, Sinclair, & Sherwin, 2009) and provide critical information on management and prevention of future invasion (Signorile et al., 2014; Veale et al., 2013; Zhang, Edwards, Kang, & Fuller, 2014). Multiple introductions, secondary expansion of introduced populations and control efforts can all lead to structured populations in introduced regions (Berthouly‐Salazar et al., 2013; Cao, Wei, Hoffmann, Wen, & Chen, 2016; Konecny et al., 2013), while gene flow between introduced populations eventually decreases levels of differentiation (Tsuchida, Kudo, & Ishiguro, 2014) except at loci under selection.

The western flower thrips (WFT), *Frankliniella occidentalis* (Pergande) (Thysanoptera: Thripidae), is a global invasive pest causing serious damage to many crops (Childers, 1997; Rotenberg, Jacobson, Schneweis, & Whiffleld, 2015). It originated in southwestern USA (Morse & Hoddle, 2006) and has invaded other areas around the world since the late 1970s (Kirk & Terry, 2003). WFT was introduced into the eastern states of USA and Canada before 1995, most states in Australia before 1996, nearly all countries across Europe before 1998, Central and South America and four countries in Africa before 2001 and four countries in Asia before 1994 (Kirk & Terry, 2003). In Asia, it was first reported in Israel in 1987, peninsular Malaysia in 1989, Japan in 1990 and South Korea in 1994 (Kirk & Terry, 2003). The invasion success of WFT is likely to reflect high levels of polyphagy, resistance to most classes of insecticides and rapid population growth due to a short generation time and parthenogenetic reproduction (Gao, Lei, & Reitz, 2012). Establishment and expansion of WFT are thought to be enhanced by rapid cropping rotations and the presence of glasshouses, which provide continuous food and habitats (Wan & Yang, 2016).

The invasion of WFT into China has occurred relatively recently. The first report of WFT establishment was in 2003 on glasshouse pepper in a suburban area of Beijing in northern China (Zhang, Wu, Xu, & Zhu, 2003). Prior to this time, WFT was first found in 2000 at an international exhibition on floriculture in Kunming, Yunnan province, in southwestern China (Jiang, Bai, Xiao, & Yang, 2001). While these locations are separated by 2,200 km, WFT began to cause heavy damage in these two regions almost simultaneously before spreading to other areas (Ren, Lei, Zhang, Hong‐Ling, & Hua, 2006; Wu, Zhang, & Li, 2009; Zhang et al., 2003; Zheng, Liu, Zhang, & Zhao, 2007). WFT has rapidly become a common pest of vegetable crops and flowers in nearly all provinces across China in 8 years since the first report of its establishment (Chen, Yuan, Du, Zhang, & Wang, 2011; Zhang et al., 2003).

While patterns of establishment of WFT in China may point to two invasions in Beijing and Yunnan, it is also possible that southwestern populations in China served as a single point of origin. Previous genetic studies based on microsatellite markers and mitochondrial variation suggested low levels of genetic differentiation of WFT among populations in China in the early phase of establishment (Yang, Sun, Xue, Li, & Hong, 2012; Yang et al., 2015). However, there is variation among Chinese WFT populations in their level of resistance to insecticides, even within the Beijing region, which points to some level of genetic differentiation among populations (Wang, Hou, 2011; Wang, Liu, et al., 2011; Wang et al., 2016).

To clarify pathways of invasion in China, we characterize the genetic diversity and population structure of WFT using new genetic markers, and we also consider variation in a population from the native range of WFT. We use 18 newly developed microsatellite loci selected by stringent criteria (Cao, Li, et al., 2016) as well as a segment of a mitochondrial gene to characterize patterns of variation. The results point to high levels of differentiation among some populations suggestive of multiple origins and long distance gene flow.

## 2 Materials and Methods

### 2.1 Sample collection and genotyping

**Figure 1:**
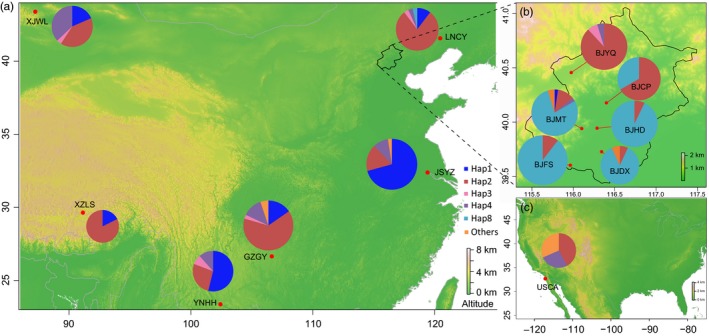
Sampling locations (red points) and mitochondrial haplotype (pie charts) distributions of *Frankliniella occidentalis* in (a) China, (b) Beijing area of China, and (c) America. Distributions of the first five dominant haplotypes are shown by different colors. The color proportions in the pie charts represent the frequency of each haplotype in a population. The size of pie charts is proportional to sample size. Codes for collection sites are shown in Table 1

We obtained WFT from 12 sites representing invaded areas in China from 2003 to 2010 when WFT became widespread (Table 1, Figure 1), as well as one site from its putative native range of California in 2005 immediately after WFT was first reported in China (Table 1). Different geographical areas were considered to investigate genetic variation in China; we sampled six sites separated by large distances (558–3,081 km) as well as six sites within the vicinity of Beijing (separated by 16–94 km). Given limited dispersal of WFT (Rhainds & Shipp, 2004) and the fact that semirural areas in Beijing are typically separated by residential housing developments, and topographical variation within the Beijing region, there is potential for a degree of isolation among WFT from the six sites sampled in the Beijing area. The thrips were collected from flowers by beating the flowers over a white enamel tray, and each thrips used in this study came from a different host plant. All specimens were preserved in absolute ethanol and stored at −80°C prior to DNA extraction. In total, 411 female WFT were used.

**Table 1:** Collection information for specimens of *Frankliniella occidentalis* used in the study

Code	Collection location	1st obs.	Longitude (°E)	Latitude (°N)	Host	Collection year	No.	Data sets
YNHH	Yunnan Province, Honghe	2005	102.4206	23.3692	Corn	2010	30	mtDNA and SSR
GZGY	Guizhou Province, Guiyang	2006	106.6302	26.6477	Chinese rose	2010	39	mtDNA and SSR
JSYZ	Jiangsu Province, Yangzhou	2010	119.4130	32.3942	Towel gourd	2010	41	mtDNA and SSR
LNCY	Liaoning Province, Chaoyang	2009	120.4504	41.5737	Hollyhock	2009	29	mtDNA and SSR
XJWL	Xinjiang Province, Urumqi	2007	87.2313	43.3899	Cucumber	2009	27	mtDNA and SSR
XZLS	Tibet Province, Lhasa	2010	91.1409	29.6456	Cucumber	2010	17	mtDNA and SSR
BJMT	Beijing, Mentougou	2005	116.1017	39.9403	Pepper	2010	32	mtDNA and SSR
BJYQ	Beijing, Yanqing, Lvfulong	2010	115.9750	40.4567	Cucumber	2010	22	mtDNA and SSR
BJFS	Beijing, Fangshan, Hancunhe	2004	115.9643	39.6020	Cucumber	2006	48	mtDNA and SSR
BJHD	Beijing, Haidian	2003	116.2872	39.9439	Spinach	2006	42	mtDNA and SSR
BJDX	Beijing, Daxing	2005	116.3414	39.7269	Cucumber	2005	29	mtDNA
BJCP	Beijing, Xiaotangshan	2004	116.3998	40.1773	Cucumber	2006	36	mtDNA
USCA	USA, California, San Diego	—	−117.1573	32.7153	Pea	2005	19	mtDNA and SSR

1st obs., year of first observation of *Frankliniella occidentalis* outbreak; No., number of individuals examined in each population; Data sets, data sets used in this study; mtDNA, mitochondrial DNA; SSR, microsatellite loci.

Genomic DNA was extracted from individuals with the DNeasy Blood and Tissue Kit (Qiagen, Germany). Eleven populations were genotyped for both nuclear and mitochondrial markers, while the other two populations from Beijing (BJDX and BJCP) were genotyped for mitochondrial variation (insufficient DNA was available from these populations for microsatellite analyses). For nuclear markers, 18 microsatellite loci designed by Cao, Li, et al. (2016) were validated in our study following the methods and criteria they proposed (Table S1). Six loci had been assigned to the stringent marker panel described in Cao, Li, et al. (2016), three had not been assigned to any panel, and nine had not been validated previously. The size of amplified PCR products was determined using an ABI 3730xl DNA Analyzer with GeneScan 500 LIZ size standard. Alleles were identified with GENEMAPPER version 4.0 (Applied Biosystems, USA).

Mitochondrial variation was characterized based on a 609‐bp DNA segment from the 5′ end of mitochondrial *cox1* gene sequenced by the primer pairs FO‐AF (5′ TTTCGTCTAACCATAAAGATATCGG 3′) and FO‐AR (5′ TAAACTTCTGGGTGCCCAAAAAATCA 3′) modified from previously published primers (Folmer, Black, Hoeh, Lutz, & Vrijenhoek, 1994) based on the mitochondrial genome sequence of WFT (Yan et al., 2012). Polymerase chain reaction (PCR) was conducted using the Mastercycler pro system (Eppendorf, Germany) under the following conditions: an initial denaturation for 2 min at 94°C, followed by 35 cycles of 10 s at 96°C, 15 s at 54°C and 1 min at 68°C, and a subsequent final extension for 10 min at 68°C. PCR components were added as recommended by the manufacturer of Takara LA *Taq* (Takara Biomedical, Japan). Amplified products were purified and sequenced directly from both strands using an ABI 3730xl DNA Analyzer by Tsingke Biotechnology Co. Ltd (Beijing, China).

### 2.2 Genetic diversity analysis

For microsatellite loci, Hardy–Weinberg equilibrium (HWE) for each locus at each site, linkage disequilibrium between each pair of loci at each site and heterozygosity excess and deficit were tested by GENEPOP version 4.2.1 (Rousset, 2008). GENCLONE version 2.0 (Arnaud‐Haond & Belkhir, 2007) was used to estimate the total number of alleles (*A*_T_) and the unbiased expected heterozygosity (*H*_E_; Nei, 1978). Furthermore, we compared the number of alleles (*A*_S_) and heterozygosity (*H*_ES_) among samples with different sample sizes using a rarefaction method in GENCLONE. Allelic richness (*A*_R_) and allelic richness of private alleles (*P*_AR_) were calculated with a rarefaction approach in HP‐RARE version 1.1 (Kalinowski, 2005) on a minimum sample size of 11 diploid individuals.

FreeNA was used to estimate the frequency of null alleles and calculate the adjusted fixation index (*F*_ST_) using the excluding null alleles correction method with 10,000 bootstraps (Chapuis & Estoup, 2007). We performed contingency tests (*G* test) for genetic differentiation (Raymond & Rousset, 1995) between population pairs with GENEPOP version 4.2.1 (Rousset, 2008).

For mitochondrial DNA, sequencing results from both strands were assembled using SEQMAN in the LASERGENE version 7.1.2 (DNASTAR, Inc., USA). Sequences of the *cox1* were aligned with CLUSTALW (Thompson, Higgins, & Gibson, 1994) implemented in MEGA version 7 (Kumar, Stecher, & Tamura, 2016). The number of polymorphic sites (*S*), haplotype diversity (*h*), and nucleotide diversity (π) were analyzed in DnaSP version 5.10 (Librado & Rozas, 2009).

### 2.3 Phylogenetic analysis

Phylogenetic relationships among the populations were inferred with POPTREE2 (Takezaki, Nei, & Tamura, 2010) using the neighbor‐joining (NJ) method (Saitou, 1987) based on microsatellite loci.

We also constructed split networks to reveal relationships among mitochondrial haplotypes using SPLITSTREE version 4.13.1 (Huson & Bryant, 2006) with the neighbor‐net method under a distance model of K2P after 1,000 bootstraps. A maximum‐likelihood phylogenetic tree was constructed based on haplotype sequences from this study and three other studies (Brunner & Frey, 2010; Rugman‐Jones, Hoddle, & Stouthamer, 2010; Yang et al., 2015) using MEGA version 7 (Kumar et al., 2016).

### 2.4 Population genetic structure

Two Bayesian cluster methods (STRUCTURE and GENELAND) and a discriminant analysis of principal components (DAPC) were used to investigate genetic structure across the populations. Population structure was first characterized with the Bayesian model‐based clustering method implemented in STRUCTURE version 2.3.4 (Pritchard, Stephens, & Donnelly, 2000). An admixture model with correlated allele frequencies was chosen. Thirty replicates for each *K* (from 1 to 10) were run with 200,000 Markov chain Monte Carlo (MCMC) iterations after a burn‐in of 100,000 iterations. The outputs of STRUCTURE were submitted to STRUCTURE HARVESTER WEB version 0.6.94 (Earl & Vonholdt, 2012) to estimate the optimal value of *K* using the Delta (*K*) method (Evanno, Regnaut, & Goudet, 2005). The membership coefficient matrices (*Q*‐matrices) of replicated runs for each *K* were combined using CLUMPP version 1.1.2 (Jakobsson & Rosenberg, 2007) with the Greedy algorithm, and then visualized using DISTRUCT version 1.1 (Rosenberg, 2004). To investigate more subtle substructuring, additional analyses were carried out on each cluster separately.

We used GENELAND to estimate the number of clusters and their spatial patterns. The analysis was conducted over 10 replicates for each *K* value (from 1 to 15) with the null allele model selected to infer the number of clusters with 1,000,000 MCMC iterations—of which every 1,000th was retained. The replicates with the highest mean logarithm of posterior probability were used to compute posterior probabilities of population membership for each pixel of the spatial domain with a burn‐in of 500.

To identify the number of different genetic clusters in the microsatellite data, DAPC was performed using adegenet version 2.0.1 (Jombart, 2008) in the R environment.

### 2.5 Isolation by distance test

Isolation by distance (IBD) was tested in samples from China by correlating pairwise genetic differentiation (estimated as *F*_ST_/(1 − *F*_ST_)) with geographical distance using the package *ade4* in R, with 10,000 permutations. Pairwise *F*_ST_ values between populations based on microsatellites were calculated with GENEPOP version 4.2.1 (Rousset, 2008), while values based on mitochondrial haplotypes were estimated with ARLEQUIN version 3.5 (Excoffier & Lischer, 2010).

### 2.6 Gene flow analysis

Recent migration rates among Chinese populations were estimated using BAYESASS 3.0.4 (Wilson & Rannala, 2003) based on microsatellite data. We performed preliminary runs (10,000,000 steps) to adjust mixing parameters for allele frequencies and inbreeding coefficients. We then conducted ten longer runs of 100,000,000 steps using different start seeds with sampling every 1,000 steps. Trace files of the ten longer runs were combined to calculate mean migration with a burn‐in of 10,000,000 using TRACER 1.6 (Rambaut, Suchard, & Drummond, 2013).

## 3 Results

### 3.1 Genetic diversity based on microsatellite loci

Significant departure from HWE was detected in seven of the 198 population–locus combinations after sequential Bonferroni correction. Multilocus tests revealed that all populations, except for GZGY and XZLS, departed significantly from HWE due to a heterozygosity deficit. Linkage disequilibrium was not detected for locus pairs across all populations. The average allelic richness was significantly different across the 10 populations from China (*F *=* *29.1, *df*1* *=* *9, *df*2* *=* *153, *p *<* *.001), varying from 3.11 in JSYZ to 6.09 in LNCY. The standardized total number of alleles (*A*_S_) ranged from 73.39 in BJFS to 171.26 in YNHH. The standardized expected heterozygosity over all loci (*H*_ES_) was between 0.466 (JSYZ) and 0.780 (XZLS; Table 1). Average allelic richness in China (A_R_ **= 5.09 on average) was significantly lower than in USA (*A*_R_ **= 10.26, *p *<* *.05). Private allelic richness was also low for the populations from China when compared to USCA (Table 2).

**Table 2:** Parameters of genetic diversity in populations of *Frankliniella occidentalis*

Population	Mitochondrial DNA					Microsatellite loci								
	*N*	*H*	*h*	π	*S*	*N*	*A*_R_	*P*_AR_	*A*_T_	*A*_S_	*H*_o_	*H*_ET_	*H*_ES_	*F*_IS_
China														
YNHH	26	4	0.643	0.00581	21	30	5.89	0.29	211	171.26	0.619	0.777	0.776	0.106
GZGY	39	6	0.561	0.00279	24	39	5.92	0.10	173	135.90	0.703	0.741	0.741	0.033
LNCY	29	5	0.369	0.00278	22	29	6.09	0.18	171	144.05	0.658	0.772	0.770	0.121
XJWL	27	4	0.687	0.00362	21	27	5.78	0.18	173	146.01	0.667	0.74	0.740	0.055
XZLS	17	2	0.309	0.00051	1	17	5.64	0.19	166	166.00	0.610	0.78	0.780	0.093
BJYQ	22	3	0.325	0.00785	20	22	5.83	0.26	125	116.35	0.621	0.726	0.726	0.133
BJMT	30	6	0.363	0.00135	4	32	4.57	0.11	137	109.81	0.582	0.652	0.652	0.053
BJFS	48	2	0.191	0.00063	2	39	3.39	0.00	90	73.39	0.507	0.568	0.568	0.081
BJHD	42	2	0.136	0.00045	2	29	4.67	0.11	111	97.38	0.592	0.652	0.651	0.083
BJDX	29	4	0.259	0.00066	4	—	—		—	—		—	—	—
BJCP	36	2	0.457	0.0015	2	—	—		—	—		—	—	—
JSYZ	41	4	0.472	0.00104	3	41	3.11	0.10	105	76.12	0.402	0.468	0.466	0.065
Mean	—	—	0.398	—	—	—	5.09	0.15	146	123.63	0.596	0.688	0.687	—
America														
USCA	19	8	0.778	0.00628	29	19	10.26	3.09	287	271.47	0.752	0.904	0.904	0.141

Sample size (*N*), number of haplotypes (*H*), number of polymorphic sites (*S*), haplotype diversity (*h*), nucleotide diversity (π) in the analyzed populations; *A*_R_, average allelic richness (for 11 specimens); *P*_AR_, private allelic richness (for 11 specimens); *A*_T_, total number of alleles; *A*_S_, standardized total number of alleles for 17 specimens per samples. *H*_ET_, expected heterozygosity; *H*_ES_, standardized expected heterozygosity (for 17 specimens); *F*_IS_, inbreeding coefficient.

The average frequencies of null alleles estimated per locus (and per population) ranged from 0.0046 in S36 (0.0265 in GZGY) to 0.0852 in S61 (0.0520 in LNCY). The *F*_ST_ estimates based on the raw and corrected data for null alleles were similar (*t* test, *p *=* *.9966), indicating that null alleles did not generate bias in the estimation of *F*_ST_. Pairwise *F*_ST_ values ranged from 0.014 (XJWL vs. LNCY) to 0.310 (BJFS vs. JSYZ) with an average *F*_ST_ of 0.134 (Table S2). The average pairwise *F*_ST_ value between USCA and populations from China was 0.114, while the average *F*_ST_ among populations from China was 0.138. Contingency tests (*G* test) revealed that all pairwise *F*_ST_ estimates were significant.

### 3.2 Genetic diversity based on mitochondrial gene

We observed 18 mitochondrial haplotypes from the WFT populations (GenBank Accession no.: KJ576869–KJ576886). Eight haplotypes were observed in the USCA population, while the number of haplotypes in invasive populations ranged from 2 to 6 (Table 2). The haplotype diversity in USCA (*h *=* *0.778) was higher than in Chinese populations (ranging from 0.325 to 0.687). Haplotype 1 was common in populations from China (both in this study and Yang et al. (2015)) and California populations as documented in a previous study (Rugman‐Jones et al., 2010), but it was not found in the currently sampled USCA sample and in populations from California sampled by Brunner and Frey (2010). In our study, haplotype 2 was the most common overall (36.8% of individuals), and it was the dominant haplotype in six populations including USCA (Figure 1). Haplotype 8 was dominant in southern populations from Beijing (BJMT, BJDX, BJHD, BJCP, and BJFS) and was not found in other populations except for one individual from LNCY (Figure 1).

Five populations (USCA, GZGY, BJMT, BJDX, and JSYZ) had private haplotypes. In USCA, private haplotypes were present in 31.58% of individuals (6/19). Thirteen novel haplotypes (hap5–hap15, hap17–hap18) were identified in our samples compared with previous studies (five from USCA, two from BJMT, two from BJDX, two from GZGY, one from LNCY and one from JSYZ).

### 3.3 Phylogenetic relationships

**Figure 2:**
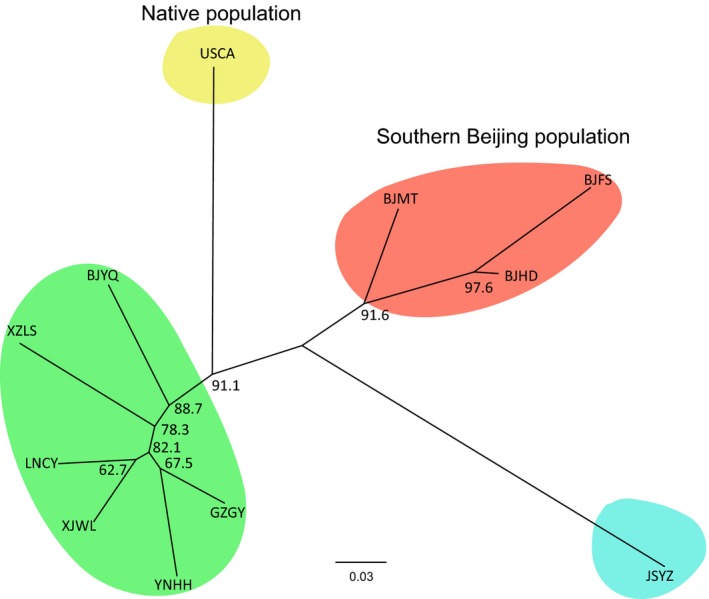
Unrooted neighbor‐joining trees for the populations constructed using microsatellite loci. Identified lineages are labeled by different color. All bootstrap values are >50%. Codes for the populations are shown in Table 1

**Figure 3:**
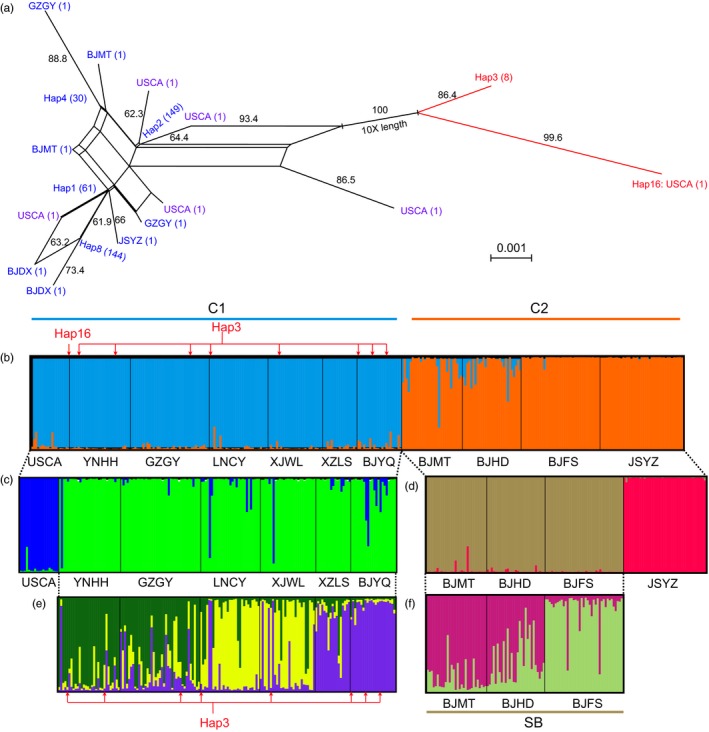
Split network for haplotypes based on mitochondrial haplotypes using SPLITSTREE (a) and nested analyses of population structure of *Frankliniella occidentalis* based on microsatellite loci using STRUCTURE (b–f). (a) Numbers in parentheses indicate the number of individuals with corresponding haplotype; three haplotypes shared by different populations were named Hap1, Hap2, and Hap4, while unique haplotypes were named by corresponding codes of population. Hap2 and Hap4 contain individuals from USCA, Hap3 is found in five populations from China, and Hap8 is mainly from five Beijing populations. Bootstrap values higher than 60 calculated from 1,000 replicates are shown near the corresponding branch. (b–f) Population structure of *F. occidentalis,* using (b) the whole data set (*N* = 322), (c) cluster C1 in “b” (*N* = 183), (d) cluster C2 in “b” (*N* = 139), (e) Chinese populations of cluster C1 in “b” (*N* = 164) and (f) southern Beijing populations (SB) of cluster C2 in “b” (*N* = 98). Codes of populations are shown in Table 1. The red arrows in “b” point to individuals with Hap3 or Hap16

In an unrooted NJ tree constructed using the microsatellite loci (Figure 2), populations from California, JSYZ, southern Beijing and other sites from China formed four distinct lineages, supported by high bootstrap values. When we analyzed mitochondrial sequences, five populations from southern Beijing formed a lineage, while the other populations formed another lineage (Fig. S1). Both split network (Figure 3a) and maximum‐likelihood trees of the mitochondrial haplotypes (Fig. S2) revealed two lineages with high bootstrapping support.

### 3.4 Population structure

The first STRUCTURE analysis using the whole data set had an optimal value of *K *=* *2 (Figure 3b). The sample from California (USCA) combined with six populations from China formed cluster 1 (C1), while the other populations formed cluster 2 (C2). When the two clusters were analyzed separately, C1 and C2 showed subdivision into two subgroups. Within C1, USCA was separated from the populations from China (Figure 3c). Within C2, JSYZ was separated from the southern Beijing populations (SB, Figure 3d). Additional analysis on populations from China in C1 revealed further subdivision into three subgroups. Samples from BJYQ and XZLS formed one cluster (blue‐purple cluster). Most samples from YNHH belonged to a separate cluster (dark green cluster). Samples from LNCY clustered with XJWL (yellow cluster). GZGY samples were particularly diverse and included individuals from all three clusters. Additional analyses on three populations from southern Beijing also separated BJFS from the other two populations (Figure 3f). Although structured populations in WFT were found in China, the two distinct mitochondrial lineages could not be separated based on nuclear marker variation (Figure 3, individuals indicated by red arrows).

The GENELAND analysis based on microsatellite data inferred three distinct clusters. The first one included six populations (Fig. S3a, corresponding to C1 in the STRUCTURE analysis), while southern Beijing populations (Fig. S3b, SB) and JSYZ (Fig. S3b) each formed a distinct cluster. There is a sharp transition from C1 to SB across a small region, which may reflect a contact zone between the two clusters.

**Figure 4:**
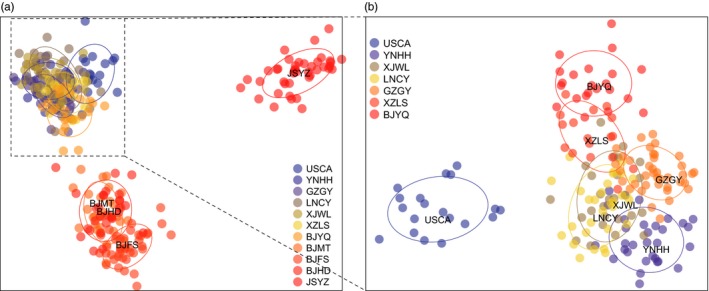
Results of a nested discriminant analysis of principal components (DAPC) showing the scatterplot of the first two principal components based on *Frankliniella occidentalis* microsatellite genotypes. (a) All individuals; the first cluster included all individuals from USCA and six populations from China (left), the second cluster consisted of all individuals from southern Beijing populations (bottom), while the third cluster included individuals from JSYZ (right). (b) Subsequent nested analysis of the third cluster of (a) distinguishing individuals from USCA and Chinese samples

The DAPC revealed similar patterns to those obtained from the STRUCTURE clustering analyses. Three clusters were identified in the first analysis (Figure 4a), consisting of USCA combined with six populations from China (C1), southern Beijing populations (BJMT, BJHD, and BJFS formed cluster SB) and JSYZ (cluster JSYZ). Samples from USCA were distinguished from the invasive populations in the subsequent nested DAPC (Figure 4b).

### 3.5 Isolation by distance

The Mantel tests based on microsatellite data sets did not reveal a significant association between genetic and geographical distances across China (*r *=* *−.050, *p *=* *.485), within the large subgroup (six populations in Figure 3e, *r* = −.163, *p *=* *.73), or within Beijing populations (*r *=* *.323, *p *=* *.263). The Mantel tests based on mitochondrial haplotypes detected a significant association within the Beijing area (*r *=* *.615, *p *=* *.03), but not across all Chinese populations (*r *=* *.104, *p *=* *.283) or in populations outside the Beijing area (*r *=* *−.055, *p *=* *.572). These results were not affected by exclusion of individuals with Hap3 and Hap16 that constituted a different mitochondrial lineage.

### 3.6 Gene flow

**Figure 5:**
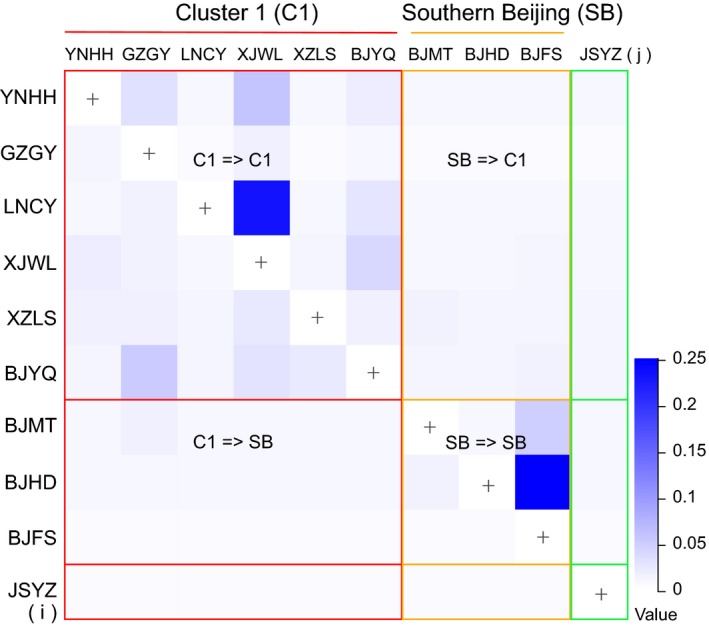
Recent gene flow among Chinese populations of *Frankliniella occidentalis* based on microsatellites data using BAYESASS. Dark color indicates high level of gene flow from population (j) to (i), while the light color indicates low level of gene flow. C1 and SB correspond to populations groups of cluster 1 and cluster of southern Beijing populations identified in STRUCTURE analyses

The estimates of gene flow obtained from the BAYESASS analysis were consistent among 10 independent runs, and mean migration rates of each population are provided as a heat map (Figure 5). A low level of gene flow was found between C1 and SB (from C1 to SB, 0.0084 on average; from SB to C1, 0.0097 on average), while a relatively higher rate of gene flow was detected within groups (within C1, 0.0255 on average; within SB, 0.0561 on average). Gene flow from or into JSYZ was estimated as consistently low.

## 4 Discussion

### 4.1 Population genetic structure of WFT after invasion

Invasive species may show genetic structure in their introduced regions, which might be caused by multiple introductions (Konecny et al., 2013), dispersal patterns following invasion (Berthouly‐Salazar et al., 2013) and/or control efforts (Cao, Wei, et al., 2016). In this study, we have used microsatellite markers and mitochondrial DNA sequences to examine the genetic structure of WFT in China. We found structured populations of WFT in the invasive area, in contrast to previous studies based on mitochondrial DNA and microsatellites isolated from an SSR‐enriched library or expressed sequence tags (Yang et al., 2012, 2015).

The reason for these inconsistent results is not entirely clear but may reflect the nature of the markers used and sampling variation. The microsatellite markers in this study were isolated from a draft WFT genome with stringent criteria, including the highest amplification rate during PCR, avoidance of single nucleotide insertions, and unambiguous peaks during genotyping, as well as the use of markers that did not deviate strongly from HWE and that were not in linkage disequilibrium. These criteria were adopted to ensure that the markers could minimize genotyping errors and reliably describe population structure, resulting in higher levels of population differentiation (*F*_ST_) and improving assignment of individuals to populations (Cao, Li, et al., 2016).

In terms of sampling sites, we included a population from eastern China (JSYZ) as well as more populations from the Beijing area where establishment of WFT was first reported, in contrast to a more limited set of locations considered in two previous studies (Yang et al., 2012, 2015). This allowed us to highlight the fact that the population from eastern China was distinct and also that the four populations from Beijing had a nested hierarchical structure and that three of these populations separated from the six other Chinese populations. Genetic differentiation was low and gene flow was high among the six geographically separate populations (YNHH, GZGY, LNCY, XJWS, XZLS, and BJYQ). This was largely consistent with previous studies that found low genetic differentiation and strong evidence for gene flow among geographically separate populations (Yang et al., 2012, 2015) that included locations near those considered here. The different conclusions of our study compared with the earlier work may therefore reflect the additional samples included in the current study.

### 4.2 Mitochondrial haplotypes

The population of WFT that has spread around the world is considered to be an insecticide resistant strain or strains from California established in glasshouses (glasshouse strain, WFTG) (Kirk & Terry, 2003). There is also evidence for a more susceptible “lupin strain” (WFTL) that spread to New Zealand prior to the spread of WFTG (Martin, Workman, & Butler, 2005; Rugman‐Jones et al., 2010). Based on mitochondrial DNA sequences and complete linkage disequilibrium between nuclear ribosomal DNA and the mitochondrial DNA, these two distinct lineages found both within and outside the native range of WFT were regarded as sympatric cryptic species by Rugman‐Jones et al. (2010). However, the two lineages have also been described as two allopatric ecotypes with distinct mitochondrial DNA and nuclear DNA sequences—a hot/dry (HD) ecotype and a cool/moist (CM) ecotype (Brunner & Frey, 2010).

The two phylogenetic lineages in this study correspond to WFTG/HD (Hap1–Hap2, Hap4–Hap15) and WFTL/CM (Hap3 and Hap16) based on mitochondrial DNA sequence variation, but we were unable to link mitochondrial DNA variation with nuclear marker variation (Figure 3). This may suggest hybridization of the two forms in China (Yang et al., 2012) in contrast to the situation in California (Brunner & Frey, 2010; Rugman‐Jones et al., 2010). Under laboratory conditions, we have observed successful hybridization of individuals from the two mitochondrial lineages sampled from field populations in China (Z.‐H. Wang, H.‐F. Jiang & S.‐J. Wei, unpublished data). To further resolve this issue, multiple samples of WFT from California should be characterized both for the microsatellite markers developed here and for mitochondrial DNA variation.

The common haplotypes found in this study were similar to those found in previous studies (Yang et al., 2015), except for haplotype 8, which was common in five populations from Beijing. This haplotype was absent from other Chinese populations (except for one individual from LNCY) or from populations in the native range of WFT that have been characterized (Brunner & Frey, 2010). However, this haplotype has previously been found in Australia (Queensland and New South Wales) and New Zealand (North Canterbury) (Rugman‐Jones et al., 2010). POPTREE analysis based on the mitochondrial gene sequences distinguished these five populations from others in China and point to five populations from Beijing having a similar origin, whereas a population from northern Beijing (BJYQ) and six other populations from China appear to have a different origin.

### 4.3 Microsatellite data

The genetic structure identified from microsatellite data, which was largely in accordance with that identified from the mitochondrial DNA variation, distinguished southern Beijing populations from others (including USCA) and supports the notion that populations in China have multiple origins. The USCA population clustered with six populations from China, and the southern Beijing populations formed another distinct cluster in both STRUCTURE analysis and DAPC. It seems possible that USCA acted as a putative source population of these six populations.

Based on historical reports, the WFT outbreak started in Beijing (in 2003) and in Yunnan Province (in 2004), with WFT becoming common in both regions by 2005 (Ren et al., 2006; Wu et al., 2009; Zhang et al., 2003). Strong genetic differentiation, distinct cluster assignments, and the substantial geographical distance between these regions (about 2,200 km) point to at least two independent introduction events of WFT into China. The source population for most Beijing populations appears different to the source for other Chinese populations, with a likely secondary introduction into Beijing. The high genetic diversity present at the YNHH site and early record of WFT at this site suggests that WFT from Yunnan served as the source population for other regions (GZGY, XZLS, XJWL, LNCY, and BJYQ) as suggested previously (Yang et al., 2012, 2015).

The southern Beijing populations and JSYZ formed two independent clusters in the DAPC and the second batch of STRUCTURE analyses. However, the POPTREE analyses based on mitochondrial DNA showed that JSYZ was distant to the southern Beijing populations but closest to YNHH. The relatively high frequency of rare alleles for some microsatellite loci in the JSYZ population may reflect an independent introduction into this region. However, founder effects can also lead to genetic differentiation between invading and/ or expanding front populations (Pierce et al., 2014; Schulte, Veith, Mingo, Modica, & Hochkirch, 2013). Founder events are particularly likely to contribute to the genetic distinctness of JSYZ given that this population was sampled as soon as it was detected and therefore prior to ongoing gene flow reducing differentiation among populations.

### 4.4 Dispersal of WFT after invasion in China

Western flower thrips across China showed no IBD even when populations with likely different origins were separately analyzed. The populations that were isolated by large distances (separated by 400–2,980 km) were clustered together (LNCY vs. XJWL, BJYQ vs. XZLS) and revealed evidence of high gene flow. These results may indicate migration of WFT mainly through human activities that span large distances. Yunnan Province is a major production area for fresh cut flowers and vegetables, and the products are transported to other regions of China and other countries (Wu, 2015; Zhu, 2015), perhaps resulting in a similar genetic composition across these regions. Beijing is an international metropolis and depends on importation of agricultural and horticultural products from other regions as well as local products. The genetic composition of WFTs within the Beijing region may partly reflect locally restricted movement patterns where vegetables and flowers are used near where they are produced.

### 4.5 Genetic diversity of WFT

Although we were unable to pinpoint the origins of Chinese populations accurately because only one sample was available from its native range, diversity levels in USCA provide a reference point for comparison. Average allelic richness and mitochondrial haplotype diversity of WFT populations in China were lower than in USCA and this may be a consequence of founder events during invasion. The average allele richness of WFT in Chinese populations was reduced to about half (60% in the case of LNCY) that found in USCA, while the average number of mitochondrial haplotypes was also reduced to 45.8% (up to 75% in GZGY and BJMT). In total, 14 mitochondrial haplotypes have been found in China (12 haplotypes in this study), accounting for 24.6% of the haplotypes found so far in WFT (Brunner & Frey, 2010; Rugman‐Jones et al., 2010; Yang et al., 2015).

Nevertheless, genetic diversity in WFT based on microsatellite markers and mitochondrial DNA was higher than in other invasive species introduced to China from America, such as the fall webworm *Hyphantria cunea* (Cao, Wei, et al., 2016), red turpentine beetle *Dendroctonus valens* (Cai, Cheng, Xu, Duan, & Kirkendall, 2008) and black locust gall midge *Obolodiplosis robiniae* (Shang, Yao, Huai, & Zhao, 2015). For instance, only one mitochondrial haplotype and an allelic richness of 2.92 were observed in 24 populations of fall webworm in China, in contrast to 12 haplotypes and an allelic richness of 6.29 in two populations from North America. The relatively high level of genetic diversity in invasive WFT populations may explain the relatively rapid development of resistance to insecticides in Chinese WFT populations (Wang et al., 2016) and other forms of local adaptation.

### 4.6 Implications for WFT management

Understanding the sources and likely dispersal pathways of invasive species is crucial for accurate risk assessment and for successful management (Stepien, Brown, Neilson, & Tumeo, 2005). Both this study and previous studies have indicated that populations from Yunnan Province may act as sources for other regions of China. Suppression and eradication of WFT populations in Yunnan are therefore likely to be important in reducing the risk of secondary expansion of this pest particularly given the likely importance of propagule pressure in the successful establishment of invasive organisms (Lockwood, Cassey, & Blackburn, 2005). Host plants of WFT, such as fresh cut flowers and vegetables, should be treated to reduce the risk of contamination by WFT when they are transported from source locations to other regions, because WFT could otherwise be moved across large distances and propagule pressure will likely increase. It is also important to prevent infected live plants being transported. Monitoring and decontamination procedures are needed not only to prevent movement of thrips between infected regions and those that are currently free of WFT. Movement of propagules from different sources could increase adaptive genetic diversity, such as by introducing new alleles resistant to pesticides, or by masking deleterious mutations when different populations have been exposed to genetic drift (Verhoeven, Macel, Wolfe, & Biere, 2011; Zidana, Turner, Van Oosterhout, & Hanfling, 2009).

## 5 Conclusion

In conclusion, we found that introduced WFT populations in China were genetically differentiated in contrast to findings from previous work. Both the mitochondrial DNA and microsatellite markers point to multiple introductions of WFT. Populations in the Beijing region arose from at least two independent introduction events, while populations in other areas likely resulted from secondary expansions out of Yunnan Province.

## Conflicts of Interest

The authors declare no conflict of interest.

## Data Archiving Statement

Data available from the Dryad Digital Repository: https://doi.org/10.5061/dryad.d3165.
