# Supplementary material for: Low genetic diversity but strong population structure reflects multiple introductions of western flower thrips (Thysanoptera: Thripidae) into China followed by human‐mediated spread
Source: Evol Appl. 2017 Feb 23;10(4):391–401. doi: 10.1111/eva.12461 (PMC5367077; doi:10.1111/eva.12461)
Supplement: Supplementary file 1 [file EVA-10-391-s001.docx]

**Supporting materials**

**Low genetic diversity but strong population structure reflect multiple introductions of western flower thrips (Thysanoptera: Thripidae) into China followed by human-mediated spread**

**Table S1** Microsatellite loci used to genotype *Frankliniella occidentalis* in this study.

**Table S2** Pairwise *F_ST_* values from comparisons between *Frankliniella occidentalis* populations based on microsatellites.

**Fig. S1** Unrooted neighbor-joining (NJ) tree drawn in POPTREE 2 using mitochondrial haplotypes.

**Fig. S2** Maximum likelihood phylogenetic tree constructed based on haplotype sequences from this study and three previous studies.

**Fig. S3** Population genetic structure of WFT inferred from GENELAND based on microsatellite data.

**Table S1** Microsatellite loci used to genotype *Frankliniella occidentalis* in this study.

| Locus | Scaffold | Motif | Forward primer 5'=>3' | Reverse primer 5'=>3' | Size (bp) | N_A_ | Tm (°C) | N |
| --- | --- | --- | --- | --- | --- | --- | --- | --- |
| wft3-S08 | KL023707 | (ACG)8 | GAAGCTGCTGTGACTCCAGT | GACGCAGAGAACGACCCTG | 152-185 | 9 | 56 | 314 |
| wft4-S09 | KL023695 | (AGGC)7 | GGCCGATGATTGTGCAAACA | CCGCATGCTAGCAATCCACT | 165-196 | 9 | 56 | 304 |
| wft3-S14 | KL023717 | (AAG)22 | TTCTCGCTCTTCAGGCGAAA | AGTATGGATTTGGCGGCGTT | 154-228 | 18 | 56 | 321 |
| wft4-S17 | KL023720 | (ACAG)20 | GACCGTCAACGTGGACCC | CCGACTGGACTGCTACTGAC | 139-271 | 25 | 56 | 315 |
| wft4-S29 | KL023712 | (ACGC)11 | CATCACGACAACAATGCCGG | AGCGTCATTATACCGGTGCC | 234-294 | 15 | 56 | 315 |
| wft4-S36 | KL023722 | (AAAG)10 | CCGGCAGCACGTTTATCAAA | TTGCGGTTGATTCGTTGCAT | 264-304 | 11 | 56 | 317 |
| wft3-S43 | KL023742 | (AGC)20 | GAGCACGCCACGATGATGAA | GACGGATGGAAGGACGCAAT | 256-384 | 22 | 56 | 322 |
| wft4-S50 | KL023714 | (ATCC)13 | CCTTGCACGCTCTGATAGGT | TCCCGTAGTTGGCCAAATGA | 295-359 | 16 | 56 | 322 |
| wft4-S58 | KL023694 | (AGAT)15 | AAGCCGAATGGGAGACACTT | ACACGTGAACAGCGTATAGGT | 338-426 | 24 | 56 | 321 |
| wft3-S61 | KL023735 | (AAC)10 | GCCACTGTTACAAACGAGCAT | CACCTTTCACGATGGCAACG | 135-175 | 13 | 56 | 322 |
| wft3-S66 | KL024032 | (AGG)11 | ATGTCATGCCGGCAGATGTA | CCGCGCACTCTCAATTTGTC | 140-198 | 18 | 56 | 319 |
| Wft4-S69 | KL023958 | (ACAG)12 | TAAGCGCTGCACTATTGGCT | CTACCGATCGACCTGCCTTG | 153-195 | 9 | 56 | 255 |
| wft3-S77 | KL023792 | (AGC)13 | GGGCTGTCTGTCTGATGTGATA | TTTCGGATTATTTCACGGGACA | 205-265 | 19 | 56 | 322 |
| wft3-S85 | KL023746 | (ACC)13 | CTGCGGACCAAAGGCTGATT | TCCAGGAGTAGACAGGGCTC | 235-308 | 17 | 56 | 322 |
| wft3-S89 | KL023805 | (AAG)13 | CGGACTCAGTTCCGACAGAG | GTCTCGCAAAGGAAACGTGG | 258-308 | 17 | 56 | 312 |
| wft3-S90 | KL024007 | (AGC)12 | TGTGGCGTCGTGGACTTTAT | AACTAGTACGTGCTGCTGGC | 232-310 | 18 | 56 | 299 |
| wft3-S93 | KL023807 | (AGC)10 | GGCTGCTCCATAATGCATGC | CACCAAGAAGGGCTGCTACA | 275-323 | 15 | 56 | 309 |
| wft3-S96 | KL024036 | (AAG)12 | AGCGGTGAATCGTGGACAAT | TTTCGGAAACACGTGGGAGG | 300-386 | 19 | 56 | 311 |

All primers were designed in [Cao et al. (2016)](#_ENREF_2). Scaffold, GenBank Accession of *Frankliniella occidentalis* genome scaffolds; Tm, annealing temperature; N_A_, number of alleles; N, number of individuals successfully genotyped from the 322 insects.

**Table S2** Pairwise *F_ST_* of *Frankliniella occidentalis* populations based on microsatellites.

| Population | USCA | YNHH | GZGY | LNCY | XJWL | XZLS | BJYQ | BJMT | BJHD | BJFS |
| --- | --- | --- | --- | --- | --- | --- | --- | --- | --- | --- |
| YNHH | 0.0864 |  |  |  |  |  |  |  |  |  |
| GZGY | 0.0734 | 0.0379 |  |  |  |  |  |  |  |  |
| LNCY | 0.055 | 0.0357 | 0.0345 |  |  |  |  |  |  |  |
| XJWL | 0.0683 | 0.0358 | 0.0419 | 0.0142 |  |  |  |  |  |  |
| XZLS | 0.0815 | 0.0636 | 0.0526 | 0.0535 | 0.0372 |  |  |  |  |  |
| BJYQ | 0.0772 | 0.0754 | 0.0339 | 0.0515 | 0.0481 | 0.0495 |  |  |  |  |
| BJMT | 0.1228 | 0.1510 | 0.1263 | 0.1249 | 0.1282 | 0.1453 | 0.1327 |  |  |  |
| BJHD | 0.1211 | 0.1443 | 0.1037 | 0.1096 | 0.1197 | 0.1330 | 0.1126 | 0.0659 |  |  |
| BJFS | 0.1893 | 0.2228 | 0.1762 | 0.191 | 0.2043 | 0.2201 | 0.1821 | 0.1171 | 0.0632 |  |
| JSYZ | 0.2638 | 0.3095 | 0.2743 | 0.2798 | 0.2856 | 0.3019 | 0.2855 | 0.2671 | 0.2771 | 0.3102 |

The values highlighted in blue are pairwise *F_ST_* values from comparisons among six populations from China that were assigned to cluster 1 in the first STRUCTURE analysis (Fig. 3b). Codes for the populations are shown in Table 1.


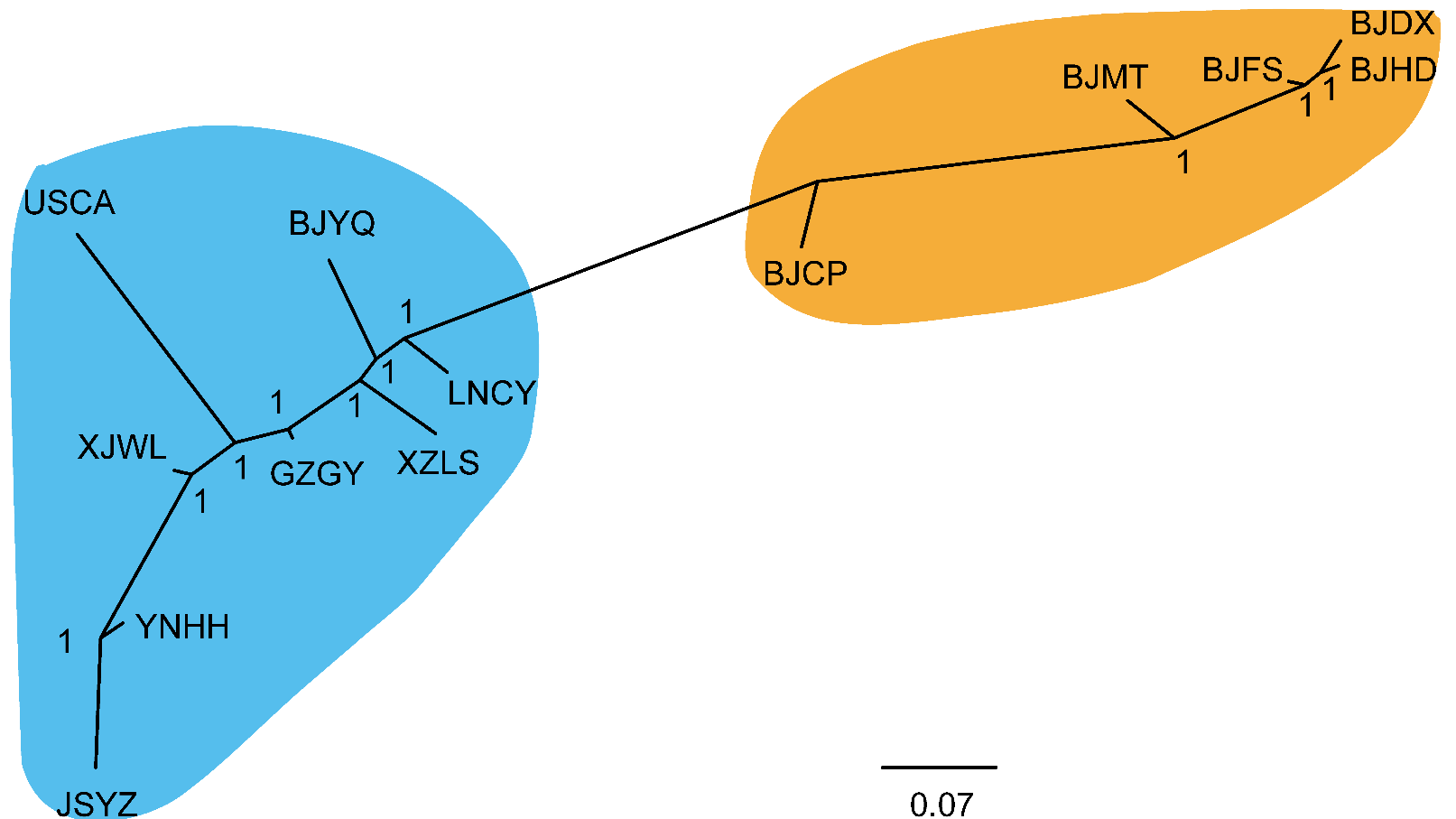


**Fig. S1** Unrooted neighbor-joining (NJ) tree drawn in POPTREE 2 using mitochondrial haplotypes. Five populations from Beijing (marked in orange) were separated from other populations. Codes for the populations are shown in Table 1.


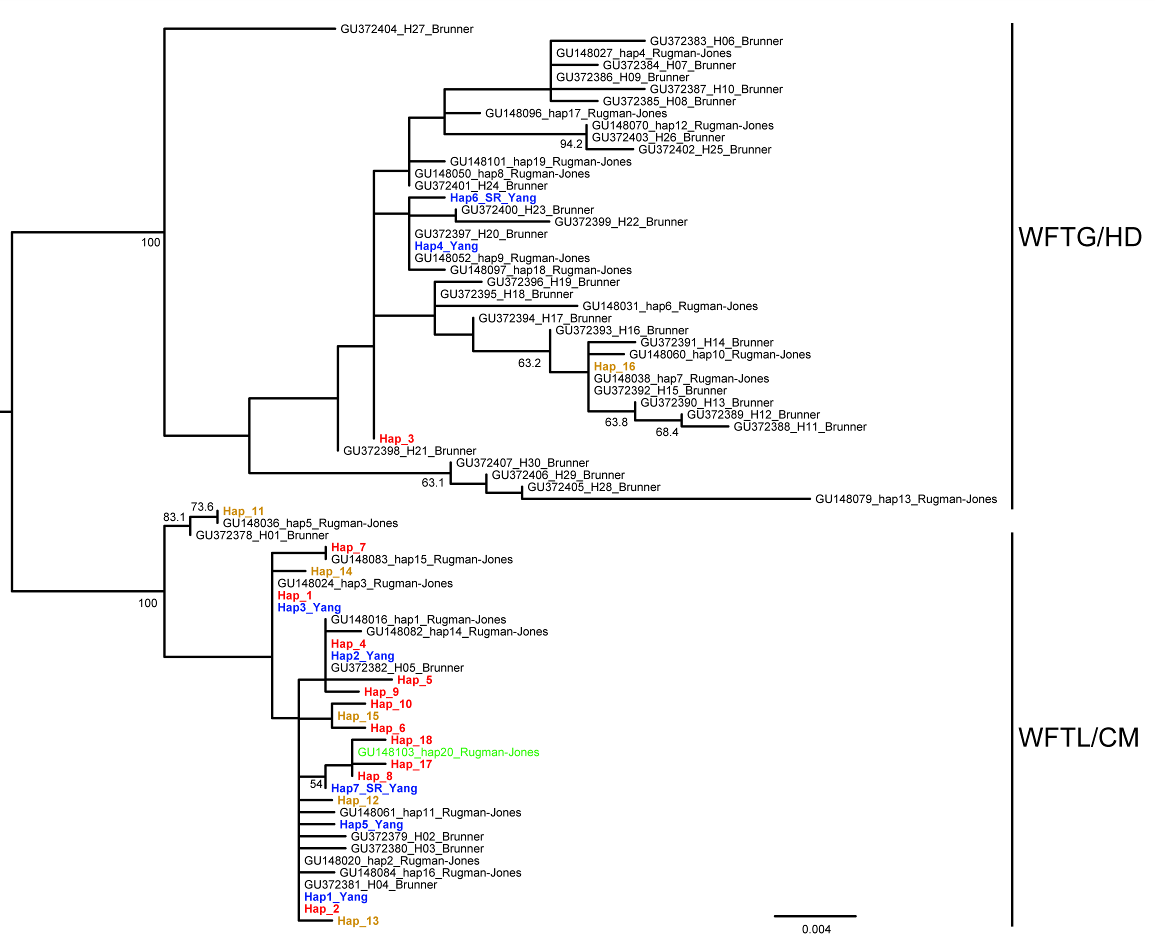


**Fig. S2** Maximum likelihood phylogenetic tree constructed based on haplotype sequences from this study and three previous studies. Red labels are haplotypes found in China in this study, blue labels are haplotypes found in China in a previous study ([Yang et al. 2015](#_ENREF_4)), brown labels are haplotypes found in USA in this study, the green label is a haplotype previously found in Australia and New Zealand, and black labels are haplotypes found in USA in a previous study. WFTG and WFTL refer to different lineages described by [Rugman-Jones et al. (2010)](#_ENREF_3), while HD and CM refer to lineages described by [Brunner and Frey (2010)](#_ENREF_1).

**
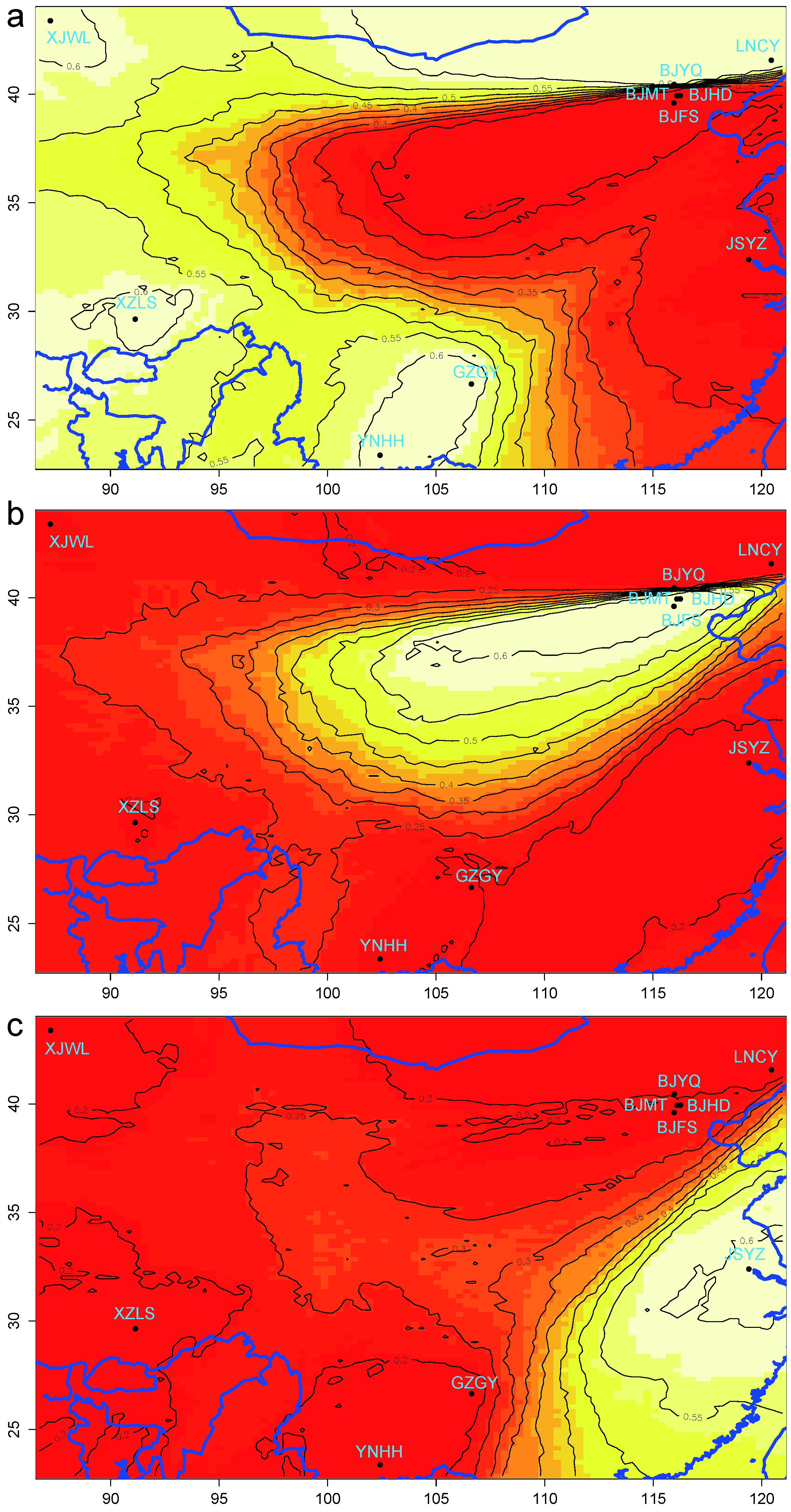
**

**Fig. S3** Population genetic structure of WFT inferred from GENELAND based on microsatellite data. Three distinct clusters were inferred (a, C1 cluster; b, SB cluster; c, JSYZ cluster). Lighter shading represents higher probability of population membership. Longitude (degrees East) and latitude (degrees North) are shown on x- and y-axes, respectively. Codes for the populations are shown in Table 1.

**References**

Brunner, P. C. and J. E. Frey. 2010. Habitat-specific population structure in native western flower thrips *Frankliniella occidentalis* (Insecta, Thysanoptera). *Journal of Evolutionary Biology* **23**:797-804.

Cao, L.-J., Z.-M. Li, Z.-H. Wang, L. Zhu, Y.-J. Gong, M. Chen, and S.-J. Wei. 2016. Bulk development and stringent selection of microsatellite markers in the western flower thrips *Frankliniella occidentalis*. *Scientific Reports* **6**.

Rugman-Jones, P. F., M. S. Hoddle, and R. Stouthamer. 2010. Nuclear-mitochondrial barcoding exposes the global pest western flower thrips (Thysanoptera: Thripidae) as two sympatric cryptic species in its native California. *Journal of Economic Entomology* **103**:877-886.

Yang, X.-M., H. Lou, J.-T. Sun, Y.-M. Zhu, X.-F. Xue, and X.-Y. Hong. 2015. Temporal genetic dynamics of an invasive species, *Frankliniella occidentalis* (pergande), in an early phase of establishment. *Scientific Reports* **5**.
